# Non-infectious risk factors for intrapartum stillbirth in a swine farm in the North of Vietnam

**DOI:** 10.14202/vetworld.2021.1829-1834

**Published:** 2021-07-16

**Authors:** Nguyen Hoai Nam, Peerapol Sukon

**Affiliations:** 1 Faculty of Veterinary Medicine, Vietnam National University of Agriculture, Trauqui, Gialam, Hanoi, Vietnam; 2 Faculty of Veterinary Medicine, Khon Kaen University, 123 Moo 16 Mittraphap Rd., Nai-Muang, Muang District, Khon Kaen 40002, Thailand; 3 Research Group for Animal Health Technology, Khon Kaen University, 123 Moo 16 Mittraphap Rd., Nai-Muang, Muang District, Khon Kaen 40002, Thailand

**Keywords:** birth weight deviation, intrapartum stillbirth, piglet, postmortem examination

## Abstract

**Background and Aims:**

Stillbirth causes considerable loss to the pig farming industry. Methods aimed at reducing stillbirth should base on the understanding of risk factors for intrapartum stillbirth because it accounts for 75% of all stillbirths. Unfortunately, few studies have differentiated between intrapartum and prepartum stillbirths leading to inadequate information about risk factors for sole intrapartum stillbirth. This study investigated risk factors for piglet’s intrapartum stillbirth.

**Materials and Methods:**

Data of 1527 piglets born from 103 sows in one herd were recorded. Generalized linear mixed models were used to determine the relationship between investigated risk factors and intrapartum stillbirth at the piglet level. The potential risk factors were parity, gestation length (GL), litter size (LS), birth order (BO), birth interval (BI), cumulative farrowing duration (CFD), gender, crown-rump length, birth weight (BW), body mass index, ponderal index (PI), and BW deviation.

**Results:**

About 60% (60.2%, 62/103) litters had stillborn piglet(s), and the intrapartum stillbirth rate was 5.8% (89/1527). BW deviation (≤0.1 and >0.6 kg), LS >13, GL (<114 and >117 days), PI ≤54, and BO >10 were the most significant factors associated with increased intrapartum stillbirth. No effect of parity, sex, BI, and CFD on intrapartum stillbirth was detected.

**Conclusion:**

These data stressed the importance of piglets’ size and shape in the prediction of intrapartum stillbirth. Furthermore, large LS, high BO, short, and long GL were associated with increased intrapartum stillbirth. The results of this study suggest that procedures aimed at increasing litter homogeneity, optimizing piglets’ size and shape, avoiding short and long gestation, and increasing supervision rate, especially at the second half, of the farrowing may reduce piglet’s intrapartum stillbirth.

## Introduction

Stillbirth accounts for 5-10% of total born piglets [1] and has been a heavy burden on modern pig farming. Increased stillbirth may be associated with various factors, including large litter size (LS), short and long gestation length (GL), long farrowing duration, and low birth weight (BW) [2]. The piglet body mass index (BMI) and ponderal index (PI) [2,3], then placental characteristics are important factors for stillbirth [3]. Furthermore, environmental factors including nutrition, farrowing induction, farrowing supervision, farrowing environment, stockmanship, stress, and drug intervention may influence the stillbirth in piglets [4].

Stillbirth was categorized into four types: Nonfresh (with a partial brown skin color due to degradation and autolysis of tissues), prepartum, intrapartum, and postpartum [5]. Non-fresh and prepartum stillbirth is mainly associated with infectious agents, whereas piglets die intrapartum and postpartum mainly due to noninfectious causes [4,5]. Therefore, studies assessing risk factors for intrapartum stillbirth should exclude the other three stillbirth types from the outcome. Unfortunately, the previous studies might include prepartum stillbirth in intrapartum since the postmortem examination was ignored [3,6-8]. Studies that might exclude prepartum stillbirth from intrapartum through postmortem examination evaluated the effect of maternal traits on intrapartum stillbirth [8,9] despite the multifactorial characteristic of stillbirth. Therefore, few studies have evaluated the simultaneous effects of both sow and piglet traits on intrapartum stillbirth in piglets [10,11].

Understanding the effects of maternal and fetal factors on intrapartum stillbirth will be helpful in establishing procedures aimed at reducing the intrapartum stillbirth rate. Therefore, this study investigated the effect of maternal and fetal factors on piglet’s intrapartum stillbirth.

## Materials and Methods

### Ethical approval

No animal samples were used in this study, and the observation and measurement were done in a humane way to avoid any stress to investigated animals.

### Study period and location

This study was conducted from July 2019 to November 2019 in a swine herd with 600 sows in the North of Vietnam.

### Animals

In total, 1527 piglets born from 103 sows of mixed parity were included in the study. All sows were Landrace×Yorkshire crossbred and were inseminated with fresh diluted semen collected from Duroc boars raised on the same farm. During gestation, sows were housed in individual crates sized 70 cm width×220 cm length. Approximately a week before the estimated farrowing date, sows were removed to individual farrowing crates sized 180 cm width×220 cm in length. At farrowing crates, sows were confined to a slatted floor area that measured 60×220 cm.

During the first 84 days of gestation, sows were fed 1.8–2.5 kg of industrial feed. Feed was increased to 3–4 kg from day 84^th^ to 110^th^ and reduced from day 111^th^ to the farrowing day at which sows were fed 1 kg. The sows’ feed contained 13–17% protein and metabolizable energy of 2900–3100 kcal/kg. Water was provided *ad libitum* through a bite nipple system.

All sows were vaccinated against classical swine fever (Coglapest, Ceva, Libourne, France), foot and mouth disease (Aftogen OLEO, Biogénesis Bagó S.A, Buenos Aires, Argentina), and porcine circovirus (Ingelvac^®^ CircoFLEX, Boehringer Ingelheim Vetmedica, Inc. Saint Joseph, MO, USA) at weeks 10, 12, and 14 of gestation, respectively. Vaccination against porcine respiratory and reproductive syndrome (Ingelvac^®^ PRRS MLV, Boehringer Ingelheim Vetmedica, Inc. Saint Joseph, MO, USA) and Aujeszky’s disease (Porcilis^@^ Begonia, Intervet, Boxmeer, Netherlands) were conducted 3 times/year. Sows were dewormed twice every year (Idectin, Thainaoka, Samut Sakhon, Thailand).

### Data collection and definition

Data were collected by two trained veterinarians. Only litters born from sows with full supervision and needed neither oxytocin nor manual extraction were included in the study. The selection strategy was based on the low incidence of oxytocin use and manual extraction in the studied farm. The parity number (PN) of all sows was recorded. GL (day) was calculated as the interval between the date of the first insemination and the date of farrowing. At the birth of each piglet, gender, birth time, and birth order (BO) were recorded. Birth interval (BI, min) was defined as the period between the births of two successive piglets. Cumulative farrowing duration (CFD, min) was calculated as the interval between the birth of a given piglet and the birth of the first piglet. Based on definitions, the first-born piglets had a CFD=0 and did not have a BI. Birth LS was calculated as the total number of born alive, stillborn, and mummified piglets. BW (kg) of piglets was measured individually using a digital scale (Weiheng, Guangzhou, China). BW deviation (BWD, kg) was the difference between the BW of a given piglet and the average BW of all stillborn and live born piglets in that litter [7]. Crown-rump length (CRL, cm) of piglet was measured using a tape measure (Garco 10, Hanoi, Vietnam). The measurement of BW and CRL lasted less than 40 s in each piglet completed within 5 min postpartum and before the colostrum intake was conducted humanely immediately after nasal and oral cavity mucous movement and body cleaning. Piglets were then put into an incubator heated with an infrared lamp. The temperature in the incubator was about 35°C. Piglets’ BMI and PI were calculated using the following equations: BW (kg)/(CRL, m)^2^ and BW (kg)/(CRL, m)^3^, respectively. All piglets that died before being put into the incubator were postmortem examined. Dead piglets were classified into five categories: (1) Mummified piglets with full brown/black skin; (2) non-fresh stillbirths with a partial brown skin; (3) prepartum stillbirths with no external sign of degeneration but showing the brick-red color of abdominal organs due to earlier degradation and autolysis of the abdominal tissues in comparison with skin [12]; the degradation and autolysis of abdominal organs suggested that these piglets died before the onset of parturition; (4) intrapartum stillbirths with standard color of abdominal organs and non-aerated lungs, and (5) postpartum deaths those died shortly after the expulsion had (partly) aerated lungs (modified from [5]).

### Statistical analysis

Descriptive statistics were derived from all available data of 103 sows and 1527 born piglets. All mummified, non-fresh, prepartum stillborn, postpartum dead, and first-born piglets, and those did not have full record were removed, leaving 1372 piglets retained in the risk analysis.

Potential risk factors for intrapartum stillbirth were PN, LS, GL, BO, BI, CFD, BW, BWD, CRL, BMI, PI, and gender. Due to potential quadratic associations between risk factors and intrapartum stillbirth, continuous independent variables were partitioned into categorical variables for the evaluation of the effect of different specific ranges of risk factors on the outcome. The categorization of the independent variables was conducted following a three-step approach using the SPSS program (IBM SPSS Statistics for Windows, Version 22.0, IBM Corp., Armonk, NY, USA). At first, the linear and quadratic relationships between each independent variable and dependent variable were examined using logistic regression. Second, the cross-tabulation with Chi-square tests was used to assess the association between a given independent variable and dependent one at different scales to detect turning points of the effect. Third, the turning points were used as cutoff values to rescale original independent variables into categorical variables.

Generalized linear mixed models (GLMMs) were used to identify risk factors for intrapartum stillbirth. The hierarchical models were built using the following format: Intrapartum stillbirth was fitted as the response, with sow fitted as a random factor to account for any potential litter differences, and other independent variables were fitted as fixed factors [13]. GLMMs began with univariate analysis to determine the most significant risk factor for intrapartum stillbirth. This factor was subsequently paired with others that were significant at p≤0.1 in the univariate analyses to be analyzed in different multivariate GLMMs. Further addition of risk factors in multivariate models was conducted on the basis of Akaike’s information criterion until the final multiple models were established. Cramer’s V coefficients were calculated to detect the potential correlations among risk factors. Marginal and conditional R-squared values were derived from measuring the percentage of variance explained by fixed factors and by entire models, respectively. Hosmer-Lemeshow goodness of fit test was conducted to determine whether the expected outcome matched the observed outcome. Model building was conducted in RStudio Desktop 1.3.1093 (RStudio Team: Integrated Development for R, Boston, MA., USA). p≤0.05 was set as the statistical significance level in the final model.

## Results

The descriptive statistics of 1527 piglets and 103 sows are presented in Table-1. Among 103 litters, 62 litters had at least one stillborn piglet (60.2%). In total, 1527 piglets were born, and 113 were stillborn (7.4%). Necropsy examination revealed that nine (8.0%) piglets were non-fresh stillbirths, ten (8.8%) piglets died prepartum, 89 (78.8%) died intrapartum, and five piglets (4.4%) died postpartum. The mummified rate was 1.4% (21/1527).

**Table-1:** Descriptive data of 103 sows and 1527 piglets.

Parameter	Mean±SD/Percentage
Sow level	
Parity	4.2±2.3
Gestation length (day)	115.7±1.7
Farrowing duration (min)	188.1±81.6
Litter size	14.8±3.1
Incidence of stillbirth at litter level (%)	60.2 (62/103)
Piglet level	
Individual birth weight^a^ (kg)	1.4±0.4
Birth interval^a^(min)	12.5± 17.8
Crown-rump length^a^(cm)	27.5±3.1
Body mass index^a^	18.4±2.8
Ponderal index^a^	68.0±15.4
Total stillbirth rate^b^(%)	7.4 (113/1527)
Non-fresh stillbirth^c^(%)	8.0 (9/113)
Prepartum stillbirth^c^ (%)	8.8 (10/113)
Intrapartum stillbirth^c^ (%)	78.8 (89/113)
Postpartum stillbirth^c^ (%)	4.4 (5/113)
Mummified rate^b^(%)	1.4 (21/1527)

a Inclusion of live and stillborn piglets;

b Inclusion of total born piglets;

c Inclusion of stillborn piglets; SD=Standard deviation

Independent variables were categorized as following: PN (1, 2–5, and 6–8), GL (112–113, 114–117, and 118–120 days), LS (5–10, 11–13, and 14–21), BO (≤5, 6–10, and 11–21), BI (≤20, 20–40, and >40 min), CFD (≤90, 90–180, and >180 min), BW (0.3–1.0, 1.0–2.1, and >2.1 kg), CRL (16–24, 24–30, and >30 cm), BMI (≤15 and >15), PI (≤54, 54–89, and >89), and BWD (<−0.5, −0.5–0.1, 0.1–0.6, and >0.6 kg). Results of univariate analyses showed that except for gender, PN, and CFD, all other factors including GL, LS, BO, CRL, BW, BMI, PI, BI, and BWD were associated with intrapartum stillbirth at p≤0.1 (Table-2). All these significant factors were further analyzed in multivariate GLMMs.

**Table-2:** Univariate and multivariate generalized linear mixed models for association between potential risk factors and intrapartum stillbirth (n=1372).

Factors	Intrapartum stillbirth rate (%)	Univariate analysis	Multivariate analysis
	
		OR; 95% CI, p	OR; 95% CI, p
PN=2–5	5.0 (36/718)	1	
PN=1	6.1 (11/181)	1.23; 0.55–2.76; 0.610	
PN=6–8	7.2 (34/473)	1.45; 0.82–2.57; 0.196	
GL=114–117 days	5.0 (54/1081)	1	1
GL=118–120 days	8.5 (14/165)	1.80; 0.88–3.70; 0.109	2.06; 1.00–4.21; 0.049
GL=112–113 days	10.3 (13/126)	2.23; 1.04–4.77; 0.040	2.36; 1.09–5.04; 0.026
LS=11–13	1.7 (3/178)	1	1
LS=5–10	3.6 (2/55)	2.22; 0.33–14.84; 0.411	3.08; 0.45–21.19; 0.252
LS=14–21	6.7 (76/1139)	4.26; 1.26–14.37; 0.019	4.26; 1.24–14.68; 0.021
F	6.3 (41/651)	1	
M	5.5 (40/721)	0.86; 0.54–1.35; 0.509	
BO≤5	4.0 (16/399)	1	1
BO=6–10	5.6 (27/482)	1.42; 0.75–2.70; 0.279	1.64; 0.85–3.16; 0.142
BO=11–21	7.7 (38/491)	2.06; 1.12–3.78; 0.021	2.26; 1.19–4.28; 0.013
CRL=24–30 cm	5.0 (53/1050)	1	
CRL>30 cm	7.6 (15/198)	1.43; 0.76–2.68; 0.264	
CRL=16–24 cm	10.5 (13/124)	2.63; 1.31–5.30; 0.007	
BW=1.0–2.1 kg	5.1 (61/1185)	1	
BW=0.3–1.0 kg	9.9 (15/151)	2.33; 1.23–4.43; 0.010	
BW>2.1 kg	13.9 (5/36)	2.65; 0.91–7.76; 0.074	
BMI>15	5.4 (70/1291)	1	
BMI≤15	13.6 (11/81)	3.15; 1.48–6.74; 0.003	
PI=54–89	5.1 (59/1165)	1	1
PI>89	9.2 (8/87)	2.22; 0.96–5.13; 0.061	1.89; 0.80–4.44; 0.145
PI≤54	11.7 (14/120)	2.45; 1.27–4.72; 0.007	2.64; 1.33–5.26; 0.006
BI≤20 min	5.4 (61/1130)	1	
BI=20-40 min	7.6 (12/158)	1.47; 0.76–2.84; 0.252	
BI>40 min	9.5 (8/84)	2.00; 0.90–4.48; 0.090	
CFD≤90 min	5.6 (38/683)	1	
CFD=90–180 min	6.0 (30/503)	1.18; 0.70–1.99; 0.527	
CFD>180 min	7.0 (13/186)	1.48; 0.72–3.05; 0.281	
BWD=0.1–0.6 kg	3.3 (16/488)	1	1
BWD=−0.5–0.1 kg	6.3 (50/789)	2.01; 1.12–3.60; 0.019	2.28; 1.25–4.14; 0.007
BWD>0.6 kg	14.8 (4/27)	4.94;1.41–17.27; 0.012	4.76; 1.34–16.85; 0.016
BWD<−0.5 kg	16.2 (11/68)	6.38; 2.71–15.02; <0.001	6.57; 2.71–15.91; <0.001

OR=Odds ratio, CI=Confidence interval, P=Probability level, PN=Parity number, GL=Gestation length, LS=Litter size, F=Female piglet, M=Male piglet, BO=Birth order, CRL=Crown-rump length, BW=Birth weight, BMI=Body mass index, PI=Ponderal index, BI=Birth interval, CFD=Cumulative farrowing duration, BWD=Birth weight deviation. The p-value, marginal R2 and conditional R2 of the final model were <0.001, 0.184 and 0.241, respectively. Hosmer-Lemeshow goodness of fit test had a p=0.175. Cramer’s V coefficients between selected independent variables ranged between 0.02 and 0.14

The most parsimonious model selected five independent variables, including BWD, PI, BO, LS, and GL, as the most significant risk factors for intrapartum stillbirth (Table-2). The final model had p<0.001. The five fixed factors and the entire model explained 18.4% and 24.1% variation of intrapartum stillbirth, respectively. Hosmer-Lemeshow goodness of fit test showed a good fit between the observed and expected outcomes (p=0.175). Cramer’s V coefficients between selected independent variables were low, ranging between 0.02 and 0.14. Piglets with a BWD of 0.1–0.6 kg had lower odds of being intrapartum stillborn than other piglets. Piglets born to a litter of 14–21 had higher odds of being intrapartum stillborn in comparison with piglets born to a litter of 11–13. A GL of 114–117 days, compared with a GL of 112–113 days and 118–120 days, reduced the odds of intrapartum stillbirth. Piglets with a PI of 54–89 had lower odds of being intrapartum stillborn, compared with piglets with a PI≤54. In comparison with BO≤5, BO above ten increased the odds of intrapartum stillbirth.

## Discussion

This study contributes to the growing knowledge of risk factors for piglet’s intrapartum stillbirth. Studies in this field did not separate prepartum stillbirth, which is generally not related to commonly investigated risk factors, from intrapartum stillbirth [3,6-8,13]. The early postmortem examination in this study reduced the possibility of misclassification of prepartum stillbirth as intrapartum stillbirth. This study indicates the existence of several risk factors of intrapartum stillbirth and highlights the importance of piglet’s body conformation traits (BW, BWD, BMI, and PI) in intrapartum stillbirth explanation.

The stillbirth rate in this study is well in a range of the previously published results [2,6,9,14-16]. Proportions of non-fresh (8.0%) and prepartum stillbirth (8.8%) in this study are in agreement with that in previous work, that is, 11.5% and 5.3%, respectively [5]. However, in this study, the proportion of intrapartum stillbirth is higher (78.8 vs. 66.8%), and that of postpartum deaths (postpartum stillbirth as defined in Leenhouwers *et al*. [5]) is lower (4.4 vs. 16.4%) [5]. A possible explanation for the discrepancy between the two studies may be attributable to the difference in the timing of necropsy examination because in that study [5] first check-up was done 12 h postpartum that might increase the incidence of postpartum death/postpartum stillbirth.

Our study confirms the previously reported results that BWD is one important indicator in explaining the variation of piglet’s intrapartum stillbirth [7]. The results indicated that piglets 0.1–0.6 kg bigger than the average BW of their litter had the lowest risk of being intrapartum stillbirth, whereas piglets smaller than the average BW of their litter and those too heavy were more likely to be stillborn. Light piglets may use oxygen less efficiently because they have lower blood hemoglobin concentration [17]. Therefore, they are more susceptible to death during the farrowing process when hypoxia is common [18]. In contrast, increased probability of intrapartum stillbirth in heavy piglets may result from an increased risk of dystocia [7]. Indeed, heavy piglets (>2.1 kg) tended to have a BI >40 min compared to medium-size piglets (19.4% *vs*. 5.2%), which may predispose them to a higher risk of intrapartum stillbirth. This study also corroborates previous studies [2,3,11,13] that PI and BMI are essential indicators of intrapartum stillbirth, but due to the high correlation between these two factors, only PI was selected in the final model. A negative linear correlation between PI and stillbirth was previously reported [3,13]. This study found that PI seemed to have a quadratic effect on intrapartum stillbirth. In comparison with normal PI piglets (PI=54–89), low PI piglets (PI≤54) tended to be smaller (20.8% *vs*. 8.9% smaller than 1 kg), and longer (61.7% *vs*. 10.6% longer than 30 cm). Therefore, piglets with a low PI were more likely to be disproportionate and may suffer some degree of intrauterine growth retardation, resulting in pathologically impaired growth [3,4,13,19], thereby acquiring a higher risk of being intrapartum stillborn.

The selection of BO as one of the most significant factors of intrapartum stillbirth rather than BI and CFD in this study agrees with the previous findings [2,3,13]. The previous reports showed that the effect of BI on stillbirth was significant only when BI exceeded 60 [20] or 90 min [21]. Therefore, the non-significant effect of BI in this study may be due to low average BI (13.4 min) and a small number of piglets with BI=60–90 min (n=28) and >90 min (n=11). Furthermore, the non-significant effect of CFD is consistent with the previous findings [22]. The explanation for the rejection of BI and CFD in favor of BO as a better indicator of intrapartum stillbirth may be linked to the fact that piglets with high BO were more likely to experience more uterine contractions. In contrast, piglets with a long CFD and BI did not necessarily share a similar condition.

An inverse association between GL and stillbirth has been reported in several studies [2,8,14,17]. In this study, the quadratic effect of GL corroborates the result of the previous studies [2,23]. The association between short GL and increased intrapartum stillbirth rate may result from the relative immaturity of piglets [17]. Alternatively, the increased intrapartum stillbirth rate of piglets born from sows with a GL of 118 to 120 days in this study may be attributable to reduced placenta’s function. Indeed, it was suggested that a prolonged gestation might cause placental insufficiency resulting in elevated stillbirth [24].

The quadratic association between LS and intrapartum stillbirth rate in this study confirms the finding of the previous study [7]. By contrast, some authors did not detect any significant effect of LS on stillbirth [3,13]. It was suggested that large LS increased intrapartum stillbirth through an inverse association between LS and BW and a direct association between LS and farrowing duration [6,9]. However, such associations were not detected in this study. Furthermore, the proportion of heavy piglets (2.1–2.5 kg) in litters of 11–13 (5.1%) was not different from that in litters of 5–10 (5.5%) and 14–21 (2.1%). The result of this study may be partly explained by the fact that piglets in litters of 11–13 tended to be born from sows with a normal GL of 114–117 days in comparison with piglets in litters of 5–10 and 14–21 (87.6% *vs*. 72.7% and 77.7%, respectively).

This study did not find any effect of parity on intrapartum stillbirth even though parity has been widely reported as a risk of intrapartum stillbirth [6,9,10]. In humans, the pressure required for cervical dilatation is higher in nulliparous parturitions than in multiparous paturitions [25]. Therefore, it might be suggested that before and during the expulsive stage piglets born from parity one sows were subjected to a higher pressure resulting in higher degrees of distress and hypoxia, which were exacerbated in smaller birth canal of parity one sows [4]. This causing a higher risk of intrapartum stillbirth in piglets born from these sows over piglets born from parity 2 – 5 sows. However, in comparison with parity 2-5 sows, parity one sows tended to have a normal GL (114-117 days, 96.7% *vs*. 76.4%), which might produce a beneficial effect to relieve the potential above-mentioned disadvantage of parity one leading to no difference in intrapartum stillbirth rate between parity one and parity 2–5. On the contrary, in this study, that piglets born from parity 2–5 sows had similar BW, BMI, PI, BI, and CFD compared with piglets born from parity 6–8 sows might result in no difference in intrapartum stillbirth rate between these two groups. Furthermore, sow selection and culling may be another possible explanation for the lack of effect of high parity on intrapartum stillbirth.

Some limitations existed in this study. First, due to the low incidence of oxytocin use and manual extraction in the studied farm, only sows without these two interventions were selected leading to failure in the evaluation of effects of oxytocin use and manual extraction, despite their potential impacts, on intrapartum stillbirth [4]. Second, necropsy might not avoid misclassification between prepartum and intrapartum stillbirths because it could not either exclude potential organ inflammation in intrapartum stillborn piglets, or detect subtle tissue degradation and autolysis in piglets that died shortly before the onset of parturition. However, in comparison with those who did not use necropsy, this study might have lowered magnitude of errors by reducing the degree of misclassification of prepartum as intrapartum stillbirths.

## Conclusion

The results of this study indicate that low and heavy BW, low PI, large LS, short and long gestation, and high BO are associated with increased intrapartum stillbirth in piglets. This data suggest that strategies increasing litter homogeneity, optimizing piglets’ size and shape, avoiding short and long gestation, and increasing supervision rate, especially at the second half, of the farrowing may reduce piglet’s intrapartum stillbirth.

## Authors’ Contributions

NHN: Collected the data. NHN and PS: Conceived and designed the study, analyzed data, interpreted results, and wrote the manuscript. Both authors read and approved the final manuscript.
